# Multidisciplinary Management of Complex Trauma and Burn Injuries: A Case Series of Challenging Clinical Scenarios

**DOI:** 10.7759/cureus.76446

**Published:** 2024-12-26

**Authors:** Selim Alameddine, Nida Khan, Sree A Purohit, Akshit Bhambri, Resheek Nerella

**Affiliations:** 1 Plastic Surgery, Beirut Arab University, Beirut, LBN; 2 Internal Medicine, Jinnah Sindh Medical University, Karachi, PAK; 3 Internal Medicine, California Institute of Behavioral Neurosciences and Psychology, Fairfield, USA; 4 Internal Medicine, Employees' State Insurance Corporation (ESIC) Medical College and Hospital, Hyderabad, IND; 5 General Surgery, University of Pennsylvania Perelman School of Medicine, Philadelphia, USA

**Keywords:** alcohol withdrawal, burn injuries, critical care, electrical burns, lightning strike, multidisciplinary management, trauma

## Abstract

Trauma and burn injuries often present with multiple complications, necessitating a coordinated, multidisciplinary approach to management. This case series reviews the outcomes and challenges of treating high-risk trauma and burn patients, with a focus on complex polytrauma, alcohol withdrawal, high-voltage electrical injuries, and lightning strikes. Each case underscores the importance of early intervention, multidisciplinary team involvement, and individualized treatment protocols for improving patient outcomes in critically injured burn victims.

## Introduction

Burn injuries, often combined with trauma, present multifaceted challenges in both acute care and long-term rehabilitation. Advances in trauma and burn care have led to improved survival rates; however, complications such as alcohol withdrawal, fluid resuscitation, and the management of electrical and lightning injuries continue to challenge medical teams.

Motor vehicle collisions (MVCs) are a leading cause of traumatic injuries worldwide, often resulting in severe polytrauma. These injuries frequently involve fractures, internal organ damage, vascular trauma, and extensive burn wounds. The combination of blunt and thermal trauma in MVC complicates clinical management, necessitating a multidisciplinary approach for optimal patient care and recovery [1].

One of the significant challenges in trauma management is the management of alcohol use disorder (AUD) in critically ill patients. Alcohol consumption significantly affects recovery outcomes, with evidence linking AUD to increased morbidity and mortality in trauma patients [2,3]. In particular, patients with alcohol withdrawal syndrome (AWS) present unique challenges, as conventional treatments, such as benzodiazepines, may not be sufficient in all cases [4]. Recent studies have emphasized alternative protocols, including the use of phenobarbital for patients unresponsive to traditional therapies [5]. A case in this case series illustrates the successful management of severe AWS in a burn patient. The patient's withdrawal symptoms were successfully managed with a continuous phenobarbital infusion, resulting in significant improvement after escalation despite initial conventional treatment.

Burn injuries, particularly electrical burns, require specialized treatment protocols distinct from those used for thermal burns. Electrical burns, while accounting for only 3-4% of burn unit admissions, often result in high morbidity and mortality rates, especially in occupational settings [6]. These injuries involve complex pathophysiology, including direct tissue destruction, thermal injury, and extensive vascular disruption, which can extend far beyond the initial burn areas [7]. Effective management requires tailored fluid resuscitation strategies and vigilant monitoring for complications such as compartment syndrome and organ dysfunction [8].

Electrical trauma also includes lightning strikes, a specific type of electrical injury with systemic effects that complicate treatment. Lightning strike injuries often result in high-intensity thermal injury, marked by severe tissue destruction and multisystem complications. Given the complexity of these injuries, they necessitate swift, multidisciplinary intervention [9]. In developing countries, electrical burns pose a substantial healthcare burden, with significantly higher mortality rates compared to other types of thermal injuries [10]. The evolving understanding of the pathophysiology of electrical burns has led to more refined treatment methods, including advanced resuscitation strategies and improved monitoring techniques.

This case series aims to showcase the diverse challenges in managing complex trauma and burn injuries, from the initial trauma phase through to recovery. By emphasizing the role of multidisciplinary collaboration and evidence-based treatment protocols, we aim to provide insight into the comprehensive management required to improve outcomes for these critically injured patients.

## Case presentation

Case 1

A 39-year-old woman was brought in after a serious MVC with an extended extrication time and numerous life-threatening injuries. Upon arrival at an outside hospital, she was found to have suffered polytrauma, including multiple fractured ribs, liver and splenic lacerations, a thoracic aortic pseudoaneurysm, complex facial and pelvic fractures, and 11% total body surface area (TBSA) burn affecting her face, chest, shoulder, and knees. Due to the severity of her injuries, an emergency splenectomy was conducted to manage the hemorrhage, after which she was transferred to our facility for specialized trauma, vascular, and burn treatment.

**Figure 1:**
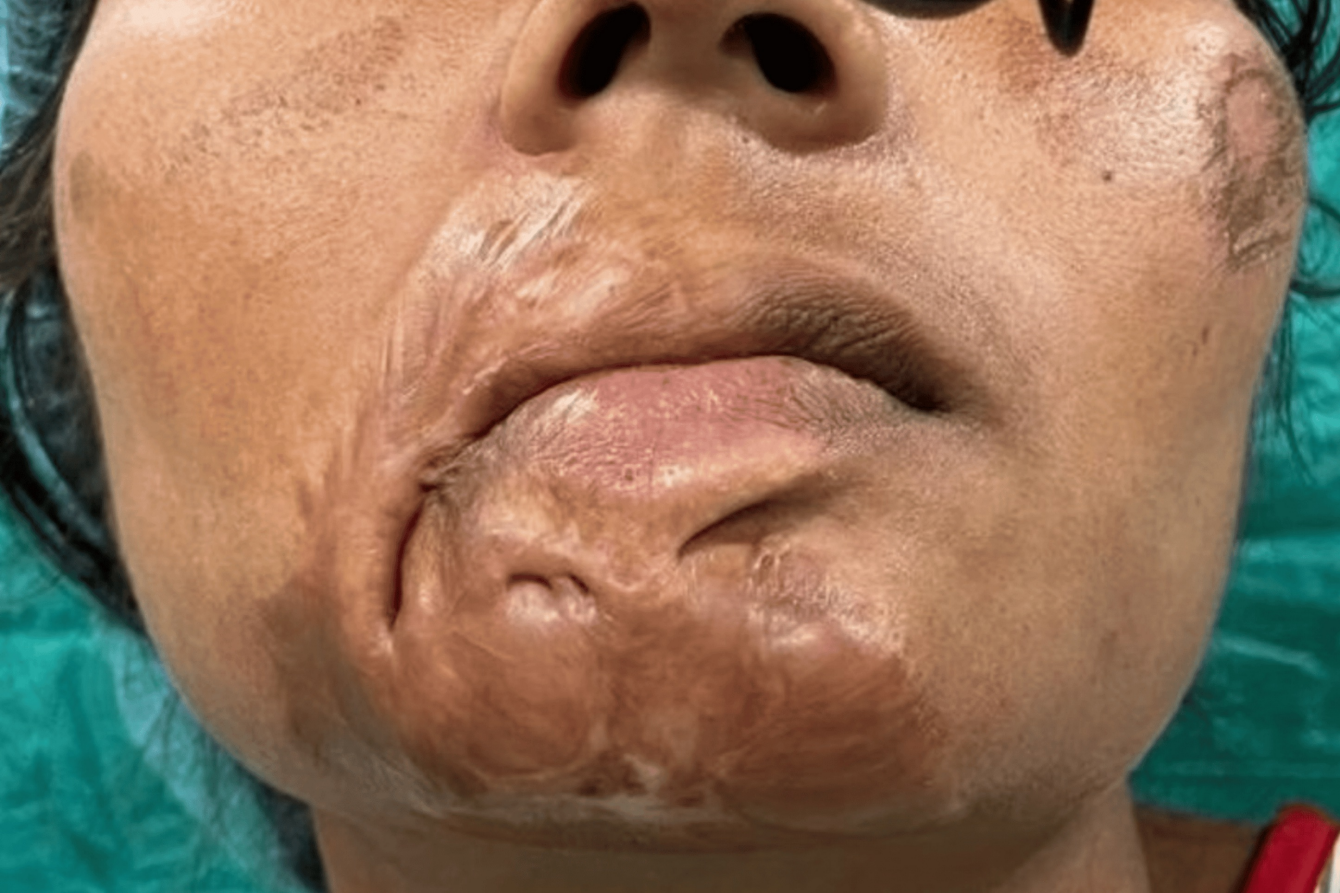
Case 1 multidisciplinary burn care for facial burns managed by a team of burn specialists

Upon her arrival, a comprehensive care team was assembled to manage her extensive injuries. Initial imaging diagnostic and clinical assessments indicated the necessity for prompt vascular treatment owing to a thoracic aortic pseudoaneurysm. A thoracic endovascular aortic repair (TEVAR) was performed in Zone 2, along with stenting of the left common carotid artery. Furthermore, surgical debridement and specialized burn care were undertaken in collaboration with burn specialists due to the patient's burns on the face, chest, and extremities, as shown in Figure 1.

Throughout her postoperative course, the patient faced several complications. Emerging infectious complications, including tracheobronchitis and Serratia bacteremia, were treated with targeted antibiotics. Despite these measures, she developed recurrent fevers, tachycardia, and increased white blood cell levels. Follow-up imaging, such as CT angiography, demonstrated a large endoleak at the previous TEVAR site, prompting immediate surgical intervention. Aortic debranching was followed by successful Zone 0 TEVAR placement.

Postoperatively, the patient developed a right-sided basal ganglia stroke, resulting in sustained neurological dysfunction. The stroke team was consulted, and neurology, physical medicine, and rehabilitation specialists ensured continuous monitoring and rehabilitation support.

Burn care continued emphasizing wound management and preventing contractures, while physical medicine and rehabilitation began early mobilization and therapeutic exercises to facilitate recovery. This patient's intricate journey emphasizes the critical role of a multidisciplinary strategy in managing polytrauma alongside concurrent burn injuries and highlights the potential complications that may arise during the post-trauma phase.

Case 2

**Figure 2:**
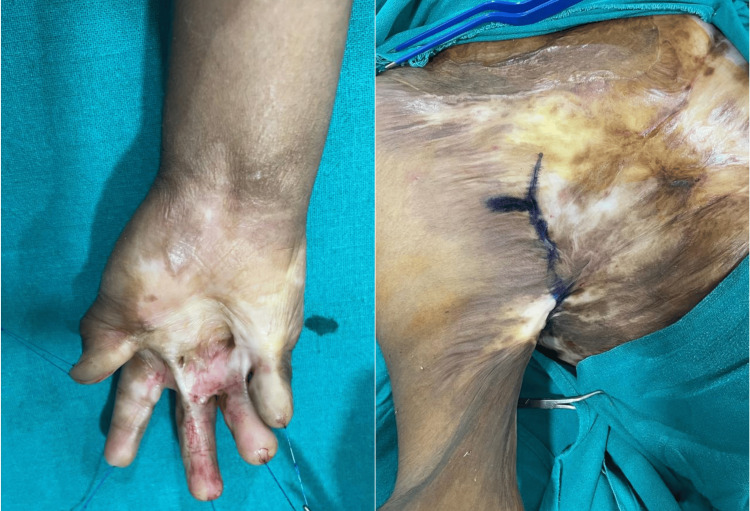
Case 2 burn injuries on the right hand and axilla after treatment is given

A 28-year-old male was brought to our burn center by emergency medical services (EMS) with second-degree burns on his right hand, right lower back, axilla, and buttocks, affecting 2.5% of TBSA. The patient was reported to have been binge drinking for three days before the injury, though the exact cause of the burns is unclear. Upon initial examination, the patient appeared obtunded with stable vital signs. Apart from the burn injuries, both the primary and secondary surveys revealed no other notable findings. Admission laboratory tests indicated an increased serum lactate of 6.1 mmol/L and a blood ethanol level of 343 mg/dL. The complete blood count, comprehensive metabolic panel, and coagulation studies showed values within the normal range. The burn injuries were managed with silver sulfadiazine dressing, and the patient was hospitalized for supportive treatment and surgical planning. The burn injuries are shown in Figure 2 after treatment is given.

After stabilization of the serum lactate and blood ethanol levels, along with cognitive recovery, burn excision and dermal substitute grafting were performed. The immediate postoperative period was stable, with manageable pain levels and adequate oral intake. However, about 24 hours postoperatively, the patient exhibited symptoms of worsening agitation, disorientation, and excessive perspiration. Lorazepam administration initially yielded short-term relief, leading to the initiation of the Clinical Institute Withdrawal Assessment (CIWA) protocol.

On the first postoperative day, the patient reported experiencing auditory and visual hallucinations. Although treated with lorazepam, diphenhydramine, and haloperidol, his symptoms escalated to profound altered mental status and tachycardia. The patient was transferred to the ICU, where a continuous phenobarbital infusion was initiated, eventually leading to significant symptom improvement. The patient was gradually weaned off dexmedetomidine and transferred back to the ward, with discharge shortly thereafter.

Case 3

**Figure 3:**
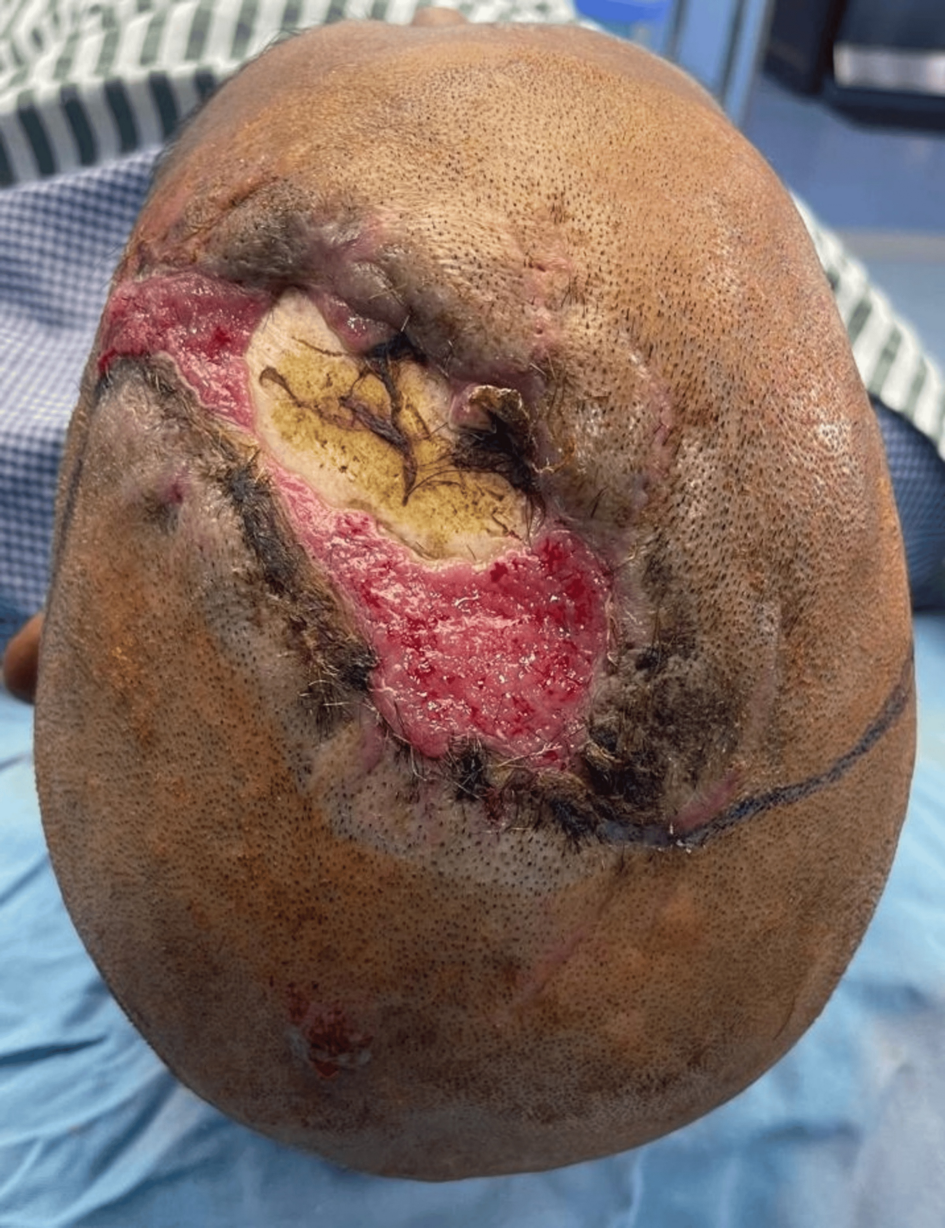
Case 3 electrothermal burns on the head following an occupational electrical injury

A 32-year-old male electrician without any significant medical history was brought to our burn center after an occupational injury while working in a cherry picker. The accident involved electrical spark exposure, leading to a combination of second- and third-degree electrothermal burns on the head, as shown in Figure 3. The initial vital signs were stable during the primary survey assessment. The patient was alert and oriented, exhibiting no signs of cognitive impairment or neurological dysfunction. An initial evaluation revealed burns covering 12% of the TBSA, mainly involving the chest and head. After hydrotherapy evaluation in the tank room, the actual TBSA was determined to be 20%. The burns exhibited varying depths: the chest was mainly affected by deep second-degree burns, while the upper extremities displayed both second- and third-degree burns.

Initial resuscitation commenced at 22:00 hours using the Burn Navigator tool (Arcos, Inc., Missouri City, TX, USA). Considering the initial estimate of direct electrical injury, fluid rates were calculated at 732 mL/hr with a target urine output of 1.0 mL/kg/hr. Throughout the acute phase, fluid needs surpassed 1L/hr at various points, prompting the use of fresh frozen plasma as a colloid supplement to sustain intravascular volume.

Further clinical assessment confirmed that direct electrical injury had not occurred, reducing the target urine output to 0.5 cc/kg/hr the next day. A retrospective review of fluid administration identified substantial volume overload resulting from the initially elevated urine output targets. To manage this, the patient was treated with furosemide to promote the elimination of excess fluid. Vital signs, urine output, and clinical parameters were monitored continuously during resuscitation. Despite the transient fluid overload, the patient stayed hemodynamically stable and did not develop complications usually associated with excessive fluid intake, including compartment syndrome or pulmonary edema. Frequent wound evaluations showed satisfactory healing, with no signs of deeper tissue involvement.

Case 4

A 29-year-old woman was brought to the emergency department by EMS following a lightning strike. She was initially discovered in cardiac arrest under a tree and was successfully resuscitated by EMS, regaining spontaneous circulation upon their arrival. The patient was subsequently intubated to ensure airway protection due to the critical nature of her condition.

Assessment in the trauma bay showed significant abnormalities with potassium levels exceeding 8 mEq/L with severe metabolic acidosis, lactate levels over 12 mmol/L, and a troponin level of 23000 ng/L. The electrocardiogram displayed ST-segment elevations. The patient was urgently transferred to the burn intensive care unit (BICU) for resuscitation, invasive line placement, and initiation of continuous renal replacement therapy (CRRT) due to severe electrolyte imbalances and acidosis.

**Figure 4:**
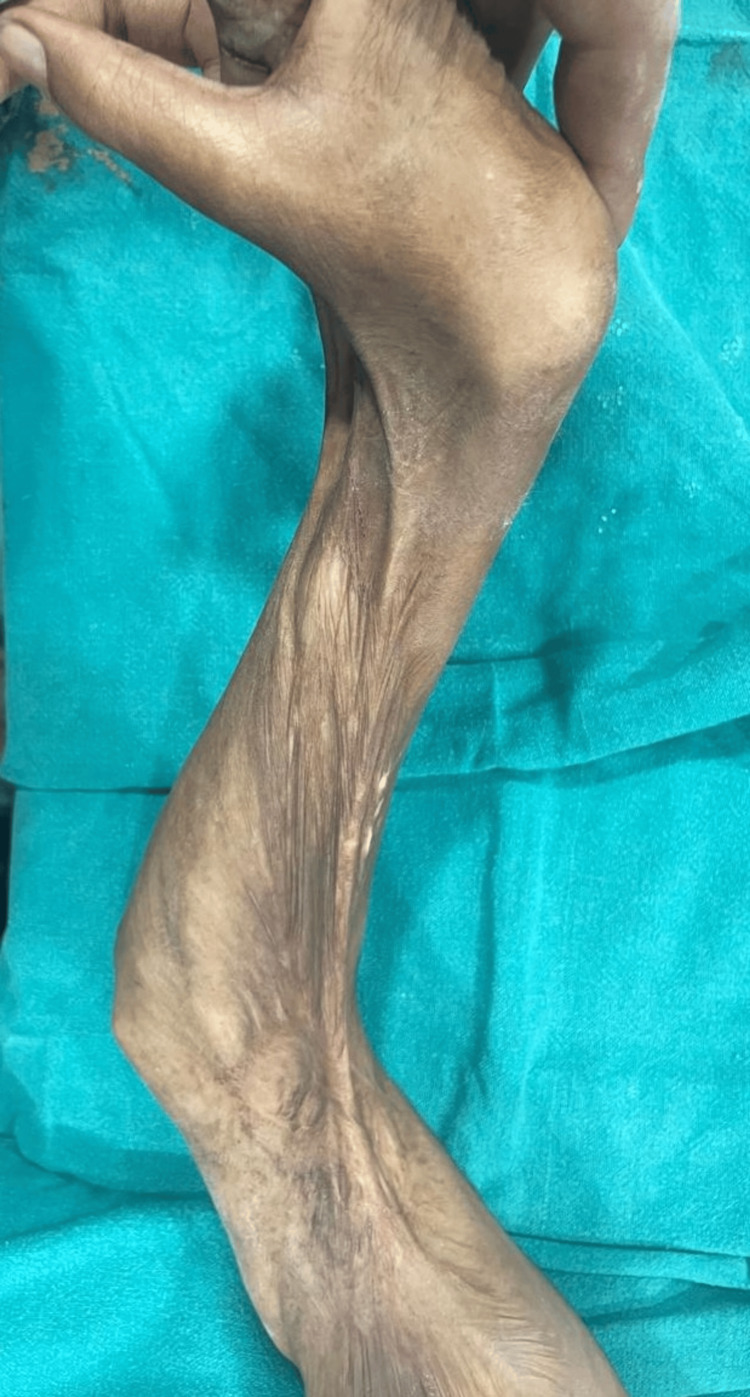
Case 4 burns contracture on the upper extremity resulting in restricted motion and skin tightening

Despite intensive care, the patient experienced a persistent hemodynamic collapse, requiring the administration of multiple vasopressors with inotropes. After multidisciplinary consultations with the BICU, cardiovascular ICU (CVICU), and cardiology teams, the patient was transferred to the CVICU for interventions such as intra-aortic balloon pump insertion and veno-arterial extracorporeal membrane oxygenation (VA-ECMO) circuit initiation. Burn contracture in the bilateral extremities was noticed, as shown in Figure 4.

Despite intensive treatment, the patient's condition continued to worsen, leading to discussions with her family regarding her prognosis and treatment preferences. After careful consideration, the family decided on comfort care, leading to the cessation of mechanical circulatory support and CRRT. The patient eventually succumbed to her injuries.

## Discussion

This case series highlights the complexity of managing severe polytrauma and burn injuries, where patients presented with diverse injuries resulting from high-impact MVCs, electrical burns, and lightning strikes. The key challenges in such cases revolve around timely intervention, addressing multiorgan injuries, and minimizing complications such as infections, electrolyte imbalances, and stroke while ensuring coordinated multidisciplinary care.

In case 1, polytrauma and severe burns from MVC, the management of polytrauma with extensive burn injuries following a high-impact MVC exemplifies the multifaceted care required. The patient, in this case, suffered severe trauma with a splenectomy performed in the initial phase of treatment, emphasizing the importance of hemorrhage control and immediate stabilization in traumatic injuries [11]. When transferred to a specialized center, advanced interventions such as TEVAR were necessary to address the traumatic aortic injury. TEVAR is considered a gold standard in managing thoracic aortic trauma due to its less invasive nature compared to open repair, but it comes with risks such as endoleak formation and neurological complications, including strokes, which require vigilant post-procedural monitoring and intervention [12,13]. In this case, the development of a stroke post-TEVAR necessitated collaboration with neurology and rehabilitation specialists, highlighting the importance of comprehensive care in polytrauma patients. The 11% TBSA burns in this case further complicated management, as burn injuries are known to trigger systemic inflammatory responses and increase susceptibility to infections such as tracheobronchitis and bacteremia [14]. Infection control, aggressive debridement, and early antibiotic therapy were crucial, but the patient still developed infectious complications, underscoring the complexity of treating burn-related infections in critically ill trauma patients.

The occurrence of endoleaks, a common complication of TEVAR, necessitated an additional procedure to correct the issue, further emphasizing the importance of early detection and management to avoid hemodynamic instability or rupture [15]. The importance of close interdisciplinary collaboration, including vascular surgeons, trauma experts, and radiologists, is evident in managing such complications. Additionally, burn injuries complicate the healing process and increase the risk of hypertrophic scarring and contractures, necessitating burn-specific rehabilitation protocols to minimize functional impairment and improve long-term recovery [16].

In Case 2, AWS in a burn patient, the second case illustrates the complex interplay between burn injuries and AWS, a challenging aspect of managing patients with burn injuries and substance abuse history. AWS occurs within 48-72 hours after the cessation of alcohol intake and is exacerbated by the stress of severe trauma and burns, increasing the metabolic demands on the body [17]. In this case, the patient’s withdrawal symptoms were severe and required escalation from standard benzodiazepine therapy to phenobarbital, which has shown efficacy in treating AWS when benzodiazepines are insufficient [18]. Recent studies support the use of phenobarbital in managing severe AWS due to its dual action on GABA and glutamate pathways, addressing both the hyperactivity of the central nervous system and autonomic instability seen in withdrawal [19,20]. Additionally, adjunctive therapies such as dexmedetomidine have been suggested to control autonomic symptoms while minimizing respiratory depression, offering another tool in the management of AWS [19].

The use of standardized assessment tools, such as the CIWA scale [20], is integral to the management of AWS; however, in burn patients, these scales may require modification due to overlapping symptoms from both pain and the body’s stress response to burn injury. Tailoring assessment protocols to specific patient populations, such as those with significant burn injuries, is an area that warrants further research. This case emphasizes the need for close monitoring and individualized treatment protocols when managing AWS in burn patients, particularly when standard treatments may be insufficient.

Case 3 highlights key aspects of modern burn care, particularly fluid resuscitation management. The initial overestimation of fluid needs, especially in suspected electrical injuries, underscores the challenges of early evaluation and the necessity for continuous reassessment of treatment strategies. Electrical burns pose distinct challenges in trauma management, necessitating specialized fluid resuscitation guidelines that vary from thermal burns. Jeschke et al. emphasized that electric burns account for just 3% to 4% of all burn unit admissions, but they are linked to high morbidity and mortality rates, especially in occupational environments [6]. Haberal et al. demonstrated that while contemporary resuscitation strategies have made significant advancements, assessing the ideal fluid needs remains challenging, particularly when the precise nature of the electrical injury is not immediately evident [21].

Recent studies, such as Cartotto and Zhou, addressed "fluid creep" - excessive fluid resuscitation - which remains a concern, especially in electrical burns where higher urine output targets are often advised [22]. The use of the Burn Navigator tool, in this case, illustrates the increasing role of computer-assisted methods in fluid resuscitation, though continuous clinical evaluation is crucial to modify protocols based on the specifics of each injury [23].

Research by Haberal et al. emphasized the importance of fluid management in the first 24-48 hours of burn resuscitation, and our case supports this, showing how initial fluid decisions can significantly affect outcomes [21]. Timely adjustments to fluid overload, as recommended by Claure et al., likely contributed to the positive result despite early over-resuscitation [24].

The distinction between direct and indirect electrical injuries, as discussed by Gentges et al., plays a critical role in shaping resuscitation strategies [25]. The patient’s recovery without complications reinforces the importance of early intervention in electrical burn cases. Overall, this case demonstrates the dynamic nature of burn resuscitation, where protocols must be adjusted based on ongoing clinical assessments. Despite initial fluid miscalculations, the outcome underscores the effectiveness of contemporary burn care protocols when coupled with careful monitoring and timely intervention [6].

In Case 4, lightning strike injury, the fourth case underscores the devastating impact of lightning strike injuries, which are characterized by a range of systemic complications, including metabolic acidosis, electrolyte imbalances, and myocardial injury. Lightning strike injuries represent a distinct type of electrical trauma that can cause various systemic complications, such as cardiac, neurological, and musculoskeletal disorders [26-30]. This patient demonstrated profound metabolic disturbances, including severe acidosis and elevated troponin levels, indicating significant cardiac injury. Lightning strikes can cause a combination of direct electrical injury and trauma from the mechanical force of the strike, leading to complex and life-threatening conditions that necessitate aggressive intervention. In this case, CRRT and mechanical circulatory support, including extracorporeal membrane oxygenation (ECMO), were required to manage severe metabolic disturbances and circulatory failure [31-33]. Despite intensive treatment, the patient’s condition continued to deteriorate, and comfort care was eventually transitioned, highlighting the high mortality associated with severe lightning strike injuries.

Managing lightning strike injuries requires early recognition of systemic complications, such as cardiac arrhythmias and rhabdomyolysis, and prompt intervention. The involvement of a multidisciplinary team with expertise in critical care, cardiology, nephrology, and burn management is crucial in optimizing outcomes [31-33]. Although ECMO and CRRT provide valuable support in managing circulatory and renal complications, the prognosis remains guarded, especially when multiple organ systems are affected. This case illustrates the need for a comprehensive understanding of the pathophysiology of lightning strike injuries and the importance of timely intervention to address the various systemic disturbances that can occur.

## Conclusions

This case series underscores the complexity and multifaceted nature of managing critically injured patients, particularly those with severe burns, polytrauma, and electrical injuries. Across the varied scenarios presented, ranging from burn resuscitation and alcohol withdrawal management to lightning strikes and electrical burn cases, several key themes emerge. First, the importance of a coordinated, multidisciplinary approach is evident in optimizing patient outcomes, particularly in addressing the unique and dynamic challenges each case presents. The cases also highlight the need for early and personalized interventions, whether in fluid resuscitation, monitoring withdrawal symptoms, or adjusting treatment protocols based on evolving clinical conditions. While contemporary burn care protocols have shown significant progress, particularly in fluid management and resuscitation strategies, these cases also reveal the need for continuous refinement and adaptability in treatment. Additionally, they emphasize the critical role of early recognition of complications, timely intervention, and the flexibility of care protocols to accommodate the individualized needs of patients. Ultimately, this series calls for continued research to refine diagnostic tools, enhance treatment algorithms, and improve predictive models for complications, particularly in high-risk burn patients, to ensure the best possible outcomes in these complex and high-acuity scenarios.
